# From Dialogue to Action: Community Recommendations for Inclusive Research Participation Among Underrepresented Populations

**DOI:** 10.1111/hex.70348

**Published:** 2025-07-30

**Authors:** Samuel Byiringiro, Grace C. Bellinger, Akunna Mezu, Erin Wong, Tosin Tomiwa, Monica Guerrero Vazquez, Adriana Foster, Payam Sheikhattari, Cheryl R. Himmelfarb, Hailey N. Miller

**Affiliations:** ^1^ School of Nursing Johns Hopkins University Baltimore Maryland USA; ^2^ School of Medicine Johns Hopkins University Baltimore Maryland USA; ^3^ School of Community Health & Policy Morgan State University Baltimore Maryland USA; ^4^ Institute for Clinical & Translational Research Johns Hopkins University Baltimore Maryland USA; ^5^ Bloomberg School of Public Health Johns Hopkins University Baltimore Maryland USA

**Keywords:** clinical research, diversity in research, equitable research, underrepresented populations

## Abstract

**Introduction:**

Clinical research is a cornerstone to medical innovation, yet Black/African American and Latino populations are underrepresented in clinical research, contributing to poorer health outcomes.

**Objective:**

This study examined perspectives on clinical research recruitment and engagement among historically underrepresented populations.

**Methods:**

Focus group discussions (FGDs) were conducted with 59 community members and leaders from Baltimore, Maryland, who identified with groups underrepresented in research. Participants were recruited via a research registry, community outreach, and word‐of‐mouth referrals. Each FGD included 8–14 participants and was conducted in English or Spanish. Thematic analysis was applied following a systematic coding process.

**Results:**

Participants included 45 (76%) community members and 14 (24%) community leaders, with 44 (75%) identifying as women, 19 (32%) as Hispanic, and 40 (68%) as Black or African American. We identified key barriers to research participation and community‐informed solutions to those barriers including: establishing community relationships to foster trust, sustaining engagement through active and transparent communication, boosting research visibility and awareness through multimodal outreach and messaging, and promoting accessibility with person‐centered accommodations. Further, we demonstrate how the proposed recommendations would help enhance the relevance, rigor, and reach of clinical research projects.

**Conclusion:**

Underrepresentation of Black or African American and Latino individuals in research hinders health equity. Findings suggest that researchers should adopt tailored strategies aligned with community needs to foster inclusive engagement.

**Patient or Public Contribution:**

The design, implementation, and interpretation of results were informed by a Community Research Council. The research council is part of a larger project which is currently implementing the recommendations from these focus group discussions. Further, research participants received a brief synopsis of the discussion for any additional feedback or comment before implementation of recommendations.

## 1 Introduction

The underrepresentation of marginalized populations in clinical research contributes to poor health outcomes and health disparities in the United States (US) [1, 2, 3, 4]. This underrepresentation has been observed in clinical research across different disease states, including cancer, diabetes, cardiovascular disease, and most recently, COVID‐19 [5, 6, 7, 8]. The inclusion and perspectives of underserved populations in the development of clinical therapies and interventions allow researchers to determine if the safety or effectiveness differs across populations. For instance, the inclusion of diverse populations allowed the discovery of the need for warfarin dosage titration in anticoagulation therapy among people from different ethnicities [9]. Not only does a lack of representation impede our ability to identify differences, but the underrepresentation of some populations has also been shown to undermine trust in the effectiveness of medical interventions for historically excluded groups [10].

Initiatives to increase representation of underserved populations in clinical research have been implemented by the National Institutes of Health (NIH) and other federal agencies. Such initiatives include the NIH Revitalization Act of 1993 and subsequent mandates to increase the inclusion of women and minorities in clinical research [11, 12] and requiring researchers conducting NIH‐defined Phase III clinical trials to submit valid subgroup analyses by sex/gender and race/ethnicity to clinicaltrials.gov [12, 13]. Recently, at the request of Congress, the National Academies of Sciences, Engineering, and Medicine (NASEM) compiled a comprehensive report on the barriers to and potential strategies for improving diverse representation in clinical trials, recognizing improvements in the representation of racial and ethnic minorities has largely stalled in recent decades [14].

The NASEM report and multiple other studies have explored barriers that contribute to the underrepresentation of underserved populations in clinical research. Historical unethical experiments, such as the United States Public Health Service Syphilis Study at Tuskegee and the unauthorized use of Henrietta Lacks' cells for research, have fueled mistrust among Black and Latino communities [15, 16]. In addition to mistrust stemming from historical events, systemic racism, discrimination in healthcare settings, and wide socioeconomic inequality further contribute to the mistrust in the medical and scientific community [17]. These are believed to contribute to lower willingness to participate in research by historically underserved populations.

The barrier of mistrust is compounded by additional factors. Black and Latino populations are less likely to receive care at clinical sites that host research studies, reducing their exposure to and engagement with such opportunities [18]. Within research institutions, limited cultural and linguistic competence among research teams can hinder their ability to understand the lived experiences and specific needs of Black and Latino populations [19]. Language barriers, combined with a lack of cultural awareness, impede meaningful communication, trust‐building, and rapport with potential participants, ultimately contributing to the continued underrepresentation of these communities in research. Given the long‐standing nature of these challenges, there is a need for specific community‐informed solutions for addressing these barriers to research participation by historically underrepresented populations. One of the key recommendations from the NASEM report calls for a paradigm shift of power from institutions and puts at the center the priorities, interests, and voices of the community [14].

Engaging all populations in research is critical for advancing the health of communities across the US. Therefore, the purpose of this study was to identify recommendations for increasing research engagement and participation among Black and Latino community members from the perspectives of residents of Baltimore, Maryland. We also explored barriers to participation to confirm alignment with previous literature and provide context for proposed recommendations.

## 2 Methods

### 2.1 Setting

The study was conducted in Baltimore, Maryland. According to the United States Census Bureau, in 2024, Baltimore had a population of 568,271 residents, and the demographic makeup of the population was approximately 60% non‐Hispanic Black or African American, 27% non‐Hispanic White, and 8% Hispanic or Latino American [20].

### 2.2 Design

The study employed a focus group discussion (FGD) design. FGDs offered participants an opportunity to express their perspectives and experiences on the barriers to participation in clinical research and communicate their recommendations for better strategies for engaging minority populations in clinical research. FGDs were conducted over three months, from August to October 2023.

### 2.3 Participants

FGD participants were adults (18 years of age or older) and able to speak English or Spanish. Community members and community leaders were the two main participant groups. Preference for participation was given to populations underrepresented in research, including Black or African American individuals and Hispanic or Latino individuals who were residents of Baltimore City. Most community leaders who participated held leadership positions in faith‐based organizations, local community‐based organizations, or research advisory groups.

### 2.4 Recruitment

Various methods of recruitment were deployed. Community leaders were recruited through individual referrals from partner organizations and community outreach specialists. Community members were recruited through outreach by attending community events and visiting community spaces, such as local markets. In the outreach events for recruitment, we offered free blood pressure screenings and various educational materials for cardiovascular disease and risk factors in both English and Spanish. Community members were further recruited by emailing over 500 participants of a registry which was established during the COVID‐19 pandemic (HOPE Registry) [21]. The algorithm used to identify people from the HOPE registry prioritized individuals that identified as Black or African American and Hispanic or Latino.

Participants were initially asked to complete an interest form administered using REDCap to collect contact information [22]. After completion, interested individuals were contacted via telephone to schedule their FGDs. To ensure consistency in communication, script dialogues were developed for both the initial phone call and follow‐up calls. Individuals who agreed to participate in a FGD were emailed a consent form and demographics survey to complete before the FGD, with the option to complete the documents during their session if preferred.

### 2.5 Focus Group Discussions

FGDs were conducted both in‐person (four sessions) and online via Zoom (three sessions), each held in the afternoon and lasting 45–60 min. In‐person discussions took place at a partnering organization's community center. FGDs were held mostly in English, with one conducted in Spanish. Accommodations, such as childcare, refreshments, and captioning, were provided to ensure ease of participation, as needed. Each FGD included a moderator, a note taker, and an observer, with confidentiality procedures and consent forms reviewed at the start. Discussions were audio recorded for data management and analysis.

While the focus of this study was to derive community recommendations for engaging underrepresented populations in clinical research, we intentionally began by discussing barriers to research participation to inform and inspire idea generation. A semi‐structured discussion guide was used to facilitate the FGDs, covering three key topics: (1) thoughts, experiences, feelings, and hesitations about participating in research; (2) ways that researchers can share information to engage people in clinical research; and (3) recruitment strategies to invite persons to participate in research, including designing culturally‐relevant materials, community outreach, and digital tools. Facilitators underwent training via Zoom to ensure consistency and familiarity with the discussion structure, fostering a collaborative environment to engage participants and generate meaningful responses. The guide, available in both English and Spanish, is provided in the supporting materials (Supporting Information S1: File 1).

### 2.6 Maintaining Rigor and Credibility

To maintain the rigor and credibility of our qualitative findings, the notetaker summarized each discussion at the conclusion of the session and asked participants whether the summary was representative of the topics and content discussed. At this time, participants were encouraged to provide any additional insights regarding barriers, facilitators, and recommendations for increasing diverse participation in clinical research.

### 2.7 Data Management and Analysis

Audio files from the FGDs were sent to a professional company for transcription. The Spanish FGD was translated from Spanish to English after transcription. Word documents were deidentified before codebook development and data analysis.

The deidentified FGD transcripts were uploaded to and organized in Dedoose software version 9.2.22 for analysis [23]. A comprehensive codebook was developed by five team members through an iterative process which involved reviewing the initial FGD guide, reading the transcripts, and pilot testing the codebook. The codebook was further presented to a larger research team for feedback before its actual utilization.

For the coding process, four investigators (GCB, AM, EW, TT) independently double‐coded the seven transcripts using the comprehensive codebook as a guide. Double‐coding was balanced, with each possible pairing coding at least one transcript. Following the initial coding, consensus coding was conducted for each transcript with a quorum being reached when the two original coders and at least one other coder were present.

Once consensus coding was complete for all seven transcripts, a list of codes was selected to export coded excerpts from Dedoose. Three team members (GCB, SB, AM) read the full dataset of relevant excerpts and grouped repeated ideas into themes and subthemes using thematic analysis.

To allow our results to inform the design and implementation of research projects, we performed an additional thematic analysis to identify community recommendations at the various stages of the research process. To accomplish this, we integrated Balazs and Morello‐Frosch's community‐based participatory research process framework which depicts how community‐engaged research can improve the relevance, rigor, and reach of research [24].

## 3 Results

A total of 59 individuals participated in the FGDs (Table 1). The highest proportion of participants (*n* = 25, 42%) were between 40 and 60 years of age and the majority identified as women (*n* = 44, 75%), non‐Hispanic (*n* = 40, 68%), and Black or African American (*n* = 40, 68%). Twenty‐seven (46%) participants were employed full‐time and about a quarter (*n* = 14, 24%) had a high school or lower level of education.

**Table 1:** Demographic characteristics for the 59 focus group discussion participants.

	Total	General community members	Community leaders
	N = 59	(*n* = 45, 76%)	(*n* = 14, 24%)
Number of focus group discussions	7	5	2
Age			
18 to < 40 years	8 (14%)	7 (16%)	1 (7%)
40 to < 60 years	25 (42%)	17 (38%)	8 (57%)
≥ 60 years	9 (15%)	7 (16%)	2 (14%)
Not reported but > 18 years	17 (29%)	14 (31%)	3 (21%)
Gender			
Man	10 (17%)	8 (18%)	2 (14%)
Woman	44 (75%)	35 (78%)	9 (64%)
Other	1 (2%)	1 (2%)	0 (0%)
Prefer not to say	4 (7%)	1 (2%)	3 (21%)
Ethnicity			
Non‐Hispanic	40 (68%)	30 (67%)	10 (71%)
Hispanic	19 (32%)	15 (33%)	4 (29%)
Race			
Black or African American	40 (68%)	31 (69%)	9 (64%)
White	7 (12%)	5 (11%)	2 (14%)
Alaska native or American Indian	2 (3%)	0 (0%)	2 (14%)
Unknown	10 (17%)	9 (20%)	1 (7%)
Employment			
Employed full‐time	27 (46%)	17 (38%)	10 (71%)
Employed part‐time	8 (14%)	7 (16%)	1 (7%)
Self‐employed	6 (10%)	5 (11%)	1 (7%)
Retired	7 (12%)	5 (11%)	2 (14%)
Unemployed	11 (19%)	11 (24%)	0 (0%)
Education			
High school or less	14 (24%)	14 (31%)	0 (0%)
Some college, associate, or bachelor's degree	23 (39%)	18 (40%)	5 (36%)
Graduate degree	22 (37%)	15 (33%)	9 (64%)

**Figure 1:**
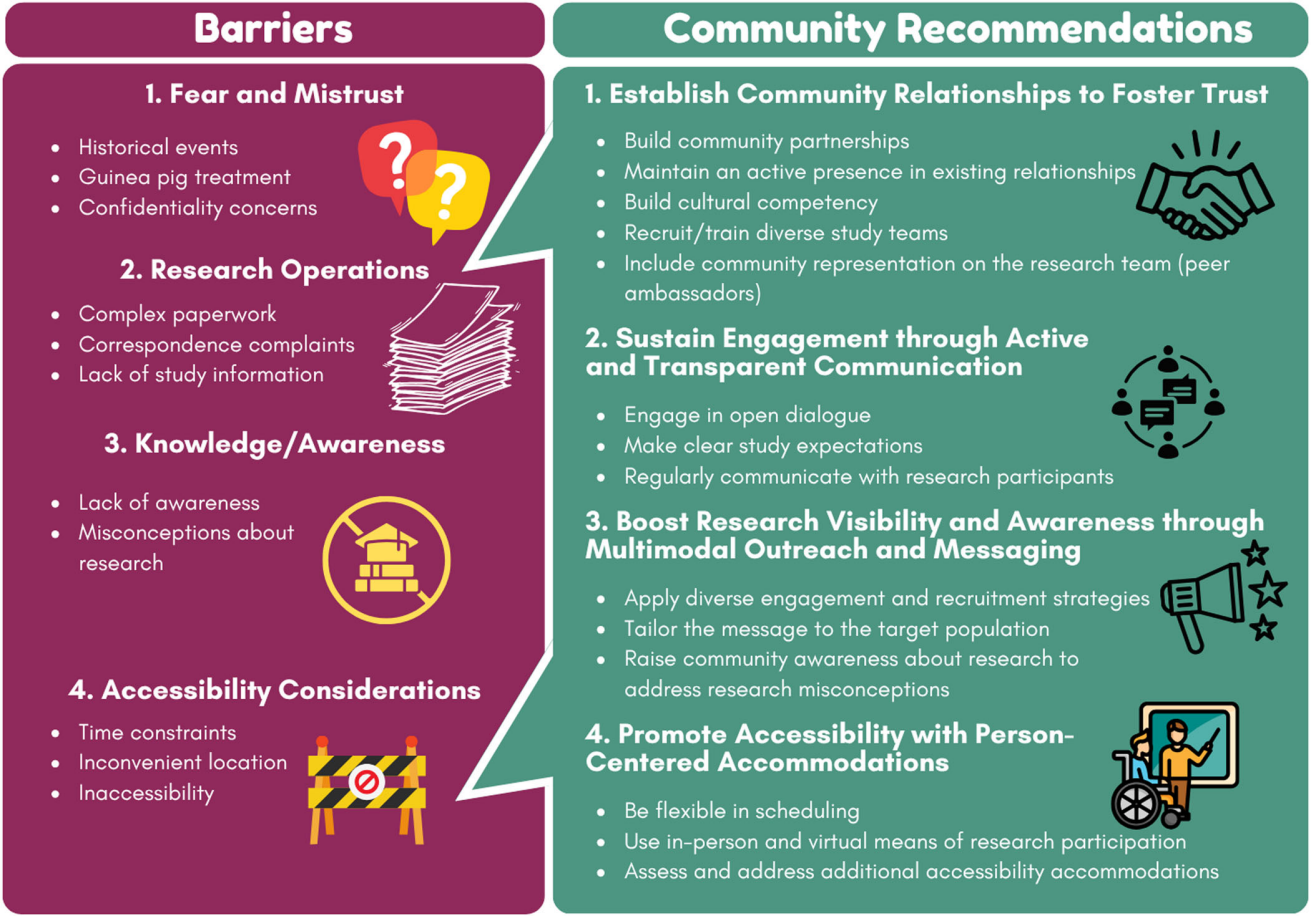
Themes ‐ barriers to and recommendations for research participation among underrepresented populations.

We found four key barriers to research participation: (1) fear and mistrust of biomedical research, (2) unfavorable research operations, (3) limited knowledge of research and awareness about research opportunities, and (4) practical and accessibility considerations (Table 2). Further, we identified four themes of recommendations: (1) establish community relationships to foster trust, (2) sustain engagement through active and transparent communication, (3) boost research visibility and awareness through multimodal outreach and messaging, and (4) promote accessibility with person‐centered accommodations. The barriers and community recommendations are illustrated in Figure 1. We further describe the subthemes for each community recommendation below using exemplar participant quotes.

**Table 2:** Barriers to research participation.

Themes	Subthemes	Quotes
**Barriers**		
Fear and mistrust	Historical events	“I think that we mentioned earlier some of the negative research that has been done even specifically on Blacks, but the Latino, the Indigenous peoples, poor people – just has been a negative. And so, when people hear about research or even see an ad to participate, there's always someone who will remind you of what has happened in the past. And you kind of get influenced by the people that you've grown up with and the community that you live in. That if they don't particularly participate in it, then you're more reluctant to participate. Like you indicated, that they go by the tales that have been told – not necessarily tales – but the stories they have been told.” (Participant 2, FGD #1)
	Guinea pig treatment	“Fear of the unknown. Because fear of being a guinea pig. Of trying something that they are not sure is safe for them. And you know you are being a guinea pig. They don't know is going to, what effect that medicine going to have on you.” (Participant 4, FGD #1)
	Confidentiality concerns	“We all have been affected by it in some way, shape or form—data breaches. I don't want my information out there… Knowing that can make people hesitant.” (Participant 1, FGD #4)
Research operations	Complex paperwork	“Streamline the enrollment process, maybe minimize some of the paperwork that—to make it easier for individuals to want to participate. Sometimes the paperwork is just too much to be a part of it. You're filling out so many things and thinking about it. You just don't have time for it.” (Participant 5, FGD #2)
	Correspondence complaints	“One big question I have—and my community has—is where does the research go, how is it being used, and how come the community never hear of the results? What happened? It kind of keeps us in the dark, which makes the community very untrustworthy of research.” (Participant 4, FGD #2)
	Lack of study information	“They invited me to a study. I got the information on MyChart. I asked the girl that sent it to me for more information. She didn't give me any information. She only told me that I could participate, that I would be rewarded—and nicely rewarded because we're talking about more than $1000… I asked her for more information, but she didn't give me any. I got scared because I mean, what if I get out of this problem worse off?” (Participant 8, FGD #5)
Knowledge/awareness	Lack of awareness	“Some individuals may not be aware of different research opportunities or how to access them, or participate them. Especially individuals in marginalized communities. Because that's sometimes the only time you see people coming around, when it comes to, ‘Hey, I need you for a research study'.” (Participant 1, FGD#2)
	Misconceptions about research	“I never saw really the need to participate in any kind of study. One because I didn't get sick or anything or had any, needed any kind of experimental drug or anything. It was…. it was not until recently something that was ever in my area of attention or my world. It was something you hear about and understood, but it really never really came across where, outside of seeing something on TV or another, where I really did not saw a role in it. Or any benefit for me in it… I really didn't see how it connected to me or anybody I cared about.” (Participant 3, FGD #1)
Accessibility considerations	Time constraints	“Time barriers. If the research project is taking place during workday or children home from school time, many reasons time is not convenient.” (Participant 3, FGD #3)
	Inconvenient location	“Someone might not have a computer if it's mobile, or if they need to go to a certain location, they might not have transportation or something like that. If they need supplies for whatever, they might not have it.” (Participant 9, FGD #3)
	Inaccessibility	“How do you communicate in an environment if you have a disability that it's hard to communicate in my case? Every time I need something, I need some sort of interpreter. If I'm going to volunteer, would they have the services in order for me to volunteer?” (Participant 4, FGD #3)

Abbreviation: FGD, focus group discussion.

### 3.1 Community Recommendation #1: Establish Community Relationships to Foster Trust

Participants strongly recommended building trusting relationships with the community to address barriers such as fear and mistrust toward clinical research. The following subthemes emerged: (a) build community partnerships, (b) maintain an active presence in existing relationships, (c) build cultural competency, (d) recruit/train diverse study teams, and (e) include community representation on the research team (peer ambassadors).

Participants emphasized that maintaining an active presence before, during, and after a research study is critical to forming meaningful relationships and can help address concerns related to mistrust. “*I think to improve… you do need to make efforts to engage the community long before you're asking them to participate in a research… I don't know that you can debunk the stories that people have heard over the years because many of them are true. But, helping people to understand that you are trying to do things differently and show proof of some of the ways you've done some things differently.” (Participant 2, FGD #1)*. In another participant's words: *“You just can't go to a community event and have an ask and that's your purpose. If that's your purpose to go with an ask for something, I think you're just wasting your time, without building relationships. But if you went to the community just to plant a seed, that may be more useful.” (Participant 3, FGD #1)*. As captured in this quote, participants felt that researchers typically only go to the community once they are actively recruiting people to join their studies and advised against this approach. Rather, respondents overwhelmingly emphasized that researchers should go to the community to build relationships before initiating research projects.

The participants recommended engaging and partnering with trusted leaders in the community from faith‐ or community‐based organizations. Specifically, several participants shared that there is an opportunity for community leaders and members to serve as ambassadors for research: “*I think that is important to work with community‐based organizations… I think that actually working a little bit more with the leaders and the teams of those organizations can yield fruitful events because they already have the trust of the community. They already have the following. If you're able to work with those leaders in a way that they can become ambassadors of what it is that you're lookin' for, that that may be helpful.” (Participant 2, FGD #4)*. Participants emphasized that including peer ambassadors brings lived experience to the study team's perspective. One participant expressed, *“…having people that look like the people you're serving as part of the research team in any way speaks volumes…—there's potentially more buy‐in, but also it reflects that you understand the community that you're serving and you are invested in them because there are people like them on your team. I think that that's really important.” (Participant 7, FGD #2)*. Participants also shared that peer ambassadors could help facilitate information sharing and enrollment: *“I think we should have peer ambassadors… They need to be focused on informing community about participating in studies. And then they can start telling people how they can register. Then know how to register when there's a study that comes that matches them, then you will have a database of who to reach.” (Participant 4, FGD #1)*.

Participants expressed several benefits to forming relationships. For example, the relationships would build a foundation of trust and facilitate researchers being viewed more positively. Specifically, one participant emphasized, “*I think it works two ways. The community and the researchers see and develop trust, and there's a partnership. Not only is there a partnership, I look at you as a real person. I can feel what you feel because I'm looking at you. You're no longer a blank piece of paper but you're a real person. I think that's what the benefits are. When a community is meeting the researchers face to face, you're viewed and looked at differently.” (Participant 2, FGD #2)*. Additionally, participants articulated that this level of engagement helps build cultural competence, ensuring research is culturally appropriate and aligns with community needs. Participant 1 said, *“…sensitivity training, really embedding that in the, the nurturing of the researcher like as a student in university, like really providing coursework that's making them more sensitive to communities… allowing them to build more trust and being more engaged, you know. Because a lot of times when you are, I've had mixed experiences around researchers, but the ones that are more like hands‐on and engaging and just, you know, talking to you like a regular kind of person, you know, I think always are just easier to warm up to, easier to trust, easier to kind of bring into the community for things like that.” (Participant 1, FGD #1)*.

### 3.2 Community Recommendation #2: Sustain Engagement Through Active and Transparent Communication

The second recommendation that emerged from the FGDs was improving communication throughout the research process. Thematic analysis revealed the following subthemes: (a) engage in open dialogue, (b) make clear study expectations, and (c) regularly communicate with research participants.

Participants recommended that researchers should clearly state the expectations of the research study upfront and discuss protocols to protect participant confidentiality and privacy, including potential uses of participant information in the future. As one participant recommended, “*Clear expectations and clear directions and with constant reminders of what the next step is going to be because sometimes you just told, ‘okay, you're gonna do this and you do that’ and then you like, ‘okay, but then what?’” (Participant 4, FGD #1)*.

While communicating with community members, participants recommended that researchers avoid scientific jargon and use simple language. This should be applied at all stages of the research process from presentation of the research study opportunity to dissemination of findings. Respondents emphasized that all information should be delivered in a language and literacy level that aligns with the community of interest. “*My perspective is to start with common language, not with that paper that has been vetted and has a lot of sentences. Try to present the purpose of the research, what you're trying to accomplish, in a colloquial way, friendly, accessible, open—present open questions that would allow the person to elaborate if something is not clear and, at a later point in that conversation, bring out the formal papers… Presenting these informed consents first is intimidating for people who are not highly educated.” (Participant 4, FGD #3)*. Importantly, participants identified that the dissemination of findings should occur within the community, in addition to scientific and academic venues. As one participant stated, “*A lot of times, once you're done with the research, you don't hear about it again; and so maintaining communication with your participants, even after the study is over, and providing them some type of updates. Once we've done this research, what are our findings? What are the outcomes? That builds trust, so if we need to go back to this community again, then the door is a little bit more open.” (Participant 5, FGD #2)*. Several participants expressed a similar sentiment: “*Send me the summary sheet so I can figure out what you got out of me.” (Participant 9, FGD #6)*.

### 3.3 Community Recommendation #3: Boost Research Visibility and Awareness Through Multimodal Outreach and Messaging

The third recommendation theme was to increase visibility of the research by using diverse outreach and messaging strategies. The subthemes for this recommendation were (a) apply diverse engagement and recruitment strategies, (b) tailor the message to the target population, and (c) raise community awareness about research to address research misconceptions.

According to research participants, the strategies for engaging with and conducting recruitment should be diversified. One participant said, *“I think using multiple modalities is going to be important because people receive and learn differently. There's gonna be folks that prefer to read some material. You're gonna have folks that prefer to see a video of it. Some people need to be tactile, hands‐on learners. How can you appeal to them?” (Participant 2, FGD #4)*. Some participants advocated for the need for researchers to get out of their institutional buildings and meet the community in the physical spaces where they naturally gather. Referring to recruitment, one participant said, *“One of the things I think you can do is that you can go where the people are. Again, many times we want to have seminars and bring people to our institutional buildings. We need to identify by building that relationship and going to where the people are. Example—there might be churches, there might be schools, community organizations. There might be the market.” (Participant 2, FGD #2)*. Additionally, participants advocated for formal use of social media for community engagement and recruitment by research institutions. A participant said, *“I think social media is a place where the name «name of a research institution» would carry weight, and so on all the crap that is out there, if I see something labeled from «name of a research institution», it would get my attention. Investing time on how to reach the people that are in social media. I'm not on there sometimes, but it's the reality for the 21st Century. Organizations like «name of a research institution» should invest in strategies, the people that know how to sell stuff to young people in social media highlighting the name that carries recognition.” (Participant 4, FGD #3)*. Participants highlighted the presence of young individuals on those platforms and potential to engage people with low literacy or disability through use of digital media for communication: *“The reason why I find that digital media is great is—it's not just, not just written, it's also little videos—is because we have a lot of people that don't know how to read or write, but then also people with disabilities.” (Participant 4, FGD #1)*.

In addition to diversifying outreach strategies, tailoring the messaging makes it resonate with the target community. It is important to note that participants, in the above quotes, reiterated the need for tailoring the message to the specific target population. A participant shared, *“Providing culturally tailored approaches… The recruitment process should be customized. That also goes along with how you're going to communicate to the individuals and making sure that you are culturally sensitive and that the research is relevant to that particular targeted population.” (Participant 5, FGD #2)*. While advocating for social media, a participant reiterated the need for tailoring the message: *“I said social media is a good way to actually share research information—because everybody's on social media—but it needs to be tailored toward that target population.” (Participant 1, FGD #1)*.

Lastly, in an effort to boost research engagement, participants recommended raising community awareness about research and addressing misconceptions—separate from study recruitment. Doing so can make people more comfortable with the word “research” and help them find their personal role in it. Here is how one participant phrased it: *“I think we want to get to the point with the word research that we're not whispering it. We're not afraid of it and we understand it. We only can do that by talking about why we research, what is research, and get it to that level where it's an open conversation—before we even invite you to be a part of a study.” (Participant 9, FGD #2)*. Another participant said, *“I think you can become more visible in the community, and just talk about it. Just talk about it and let it permeate in the air. Talk about it… Make it a household name. Make it feel like it's part of what we should do to help out.” (Participant 2, FGD #2)*.

### 3.4 Community Recommendation #4: Promote Accessibility With Person‐Centered Accommodations

To address challenges of clinical research accessibility and accommodations, participants shared recommendations in three areas: (a) be flexible in scheduling, (b) use in‐person and virtual means of research participation, and (c) assess and address additional accessibility accommodations.

Participants recommended flexible timing, as one put it, “*I think it's access to where they are asked to come, to get to a location, maybe the timeframe. I think we have to be considerate of everyone's schedule and not have a window that only suits the institution or the researcher. Have some flexibility there that sometimes interfere with an individual who might want to participate, but not be able to because of their personal schedule or needs, or accessibility to get there*.” *(Participant 2, FGD #2)*. Another encouraged researchers to “*Offer the ability to volunteer during nonbusiness hours. If I'm working 9:00 to 6:00, can I still volunteer at times that are convenient to me, not to the provider?” (Participant 4, FGD #3)*.

In addition to flexible timing, respondents also encouraged multiple participation modalities (i.e., in‐person or online) and locations (with preference to easily accessible locations). *“I agree because having this virtually like this was beneficial because running children around and everything else I have going on in life, to have to drive somewhere else was too much, so this kind of option is valuable.” (Participant 5, FGD #3)*. Participants also recommended catering support systems for people who require accommodations, such as childcare, language assistance, technological support, transportation, and parking. For every clinical research study and every eligible individual, researchers are encouraged to identify and address relevant practical barriers to participation. *“Making things convenient. Having an alternative… a plan B. If I can't come down to the center, can somebody meet me? Because you're dealing with oppressed people who have to worry about keeping the lights on or taking care of kids… But, you know, not having a cookie‐cutter approach, having a plan B, or an alternative. More than one way, if possible, to collect. You know, convenience.” (Participant 3, FGD #1)*.

### 3.5 Incorporating Community Recommendations Into the Research Process

**Figure 2:**
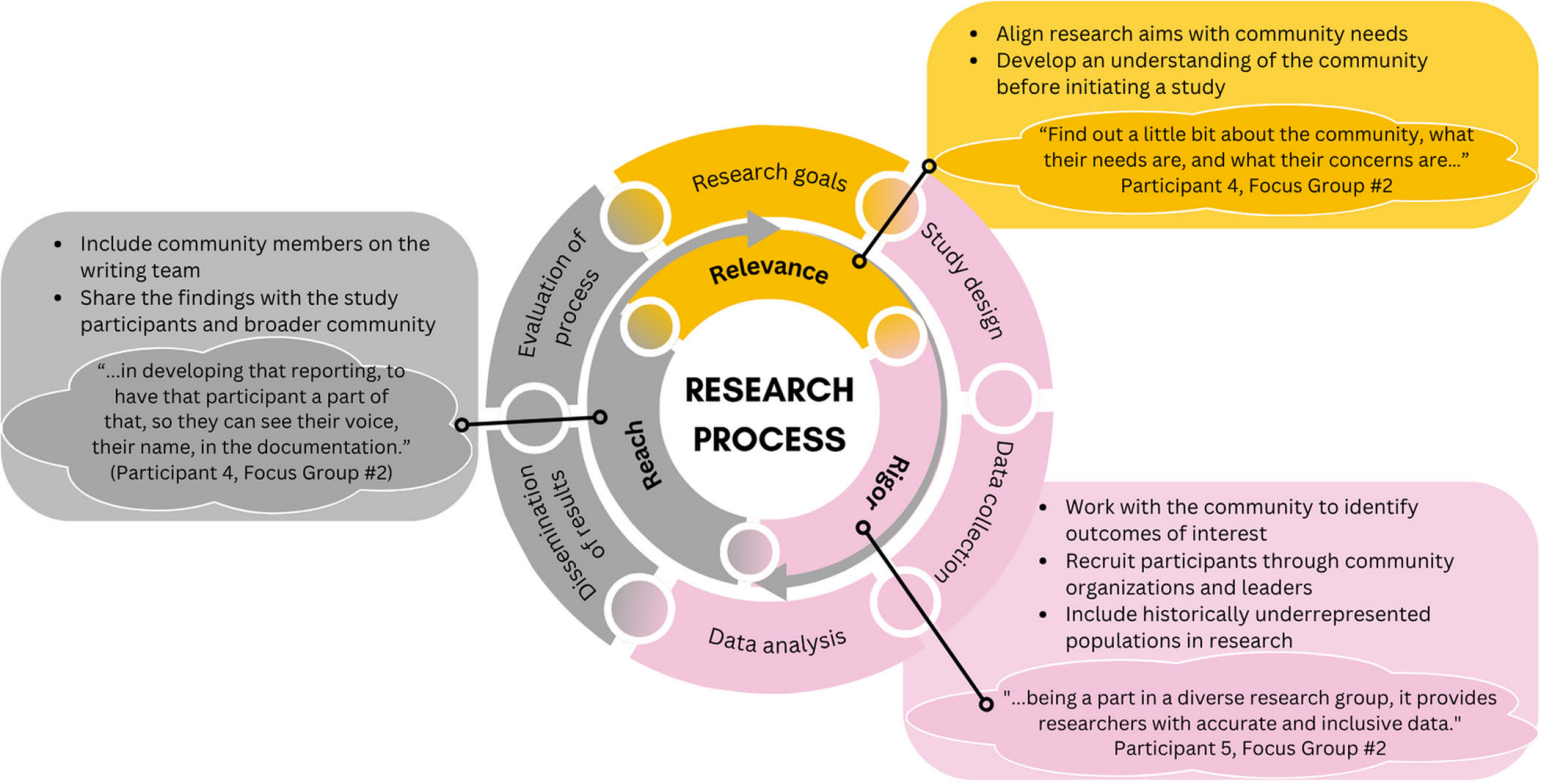
Integrating community recommendation in the research process. Notes: (1) List of applicable recommendations is not meant to be exhaustive. (2) The research process is adapted with permission from the Three R's: How Community‐Based Participatory Research Strengthens the Rigor, Relevance and Reach of Science. Reference: Balazs CL, Morello‐Frosch R. The Three R's: How Community Based Participatory Research Strengthens the Rigor, Relevance and Reach of Science. Environ Justice. 2013 Feb;6(1):10.1089/env.2012.0017. doi: 10.1089/env.2012.0017. (3) Additional representative quotes for each step of the research can be found in the Supporting Information S2: Table S1.

The community recommendations associated with each stage of the research process are provided in Figure 2. To promote the relevance of the research at the goal setting and study design stages, the researchers should develop an understanding of the community before study initiation and align the research aims with the needs of the community. Further, to ensure rigor in the design and implementation (data collection and analysis) of the study, researchers should collaborate with community members and leaders to develop protocols and ensure diversity in the recruited participants. Finally, to increase the reach, participants recommended community representation in the dissemination process (e.g., writing teams) and going back to the community to disseminate the results. We provide supporting quotes from our results for each step of the research process in Supporting Information S2: Table S1.

## 4 Discussion

This study aimed to identify strategies for increasing research participation among Black and Latino populations. In our thematic analysis, four key barriers to participation emerged: fear and mistrust of biomedical research, unfavorable research operations, limited knowledge of research and awareness about research opportunities, and practical challenges with accessibility of research. To address these barriers, participants recommended establishing community relationships to foster trust, sustaining engagement through active and transparent communication, boosting research visibility and awareness through multimodal outreach and messaging, and promoting accessibility with person‐centered accommodations. While barriers to participation among historically underrepresented groups are well‐documented [25], our findings underscore that long‐standing and well‐documented barriers to research continue to undermine trust and contribute to hesitancy among historically underrepresented populations to participate in clinical research. Importantly, our study offers actionable, community‐informed strategies to rebuild trust and foster sustained engagement in clinical research, with the focus on potential interventions before, during, and after community recruitment for research participation.

According to participants in the current study, researchers must establish community relationships to foster trust. In a survey of 220 clinical research coordinators, 71% viewed strong relationships between clinicians and potential participants—as well as reputation of the research institution—as the most important facilitators to successful recruitment [26, 27]. While the recommendations of our study for establishing relationships to foster trust largely echoed existing literature, the strategy of partnering with lay community members (i.e., peer ambassadors) to champion the research project and support the engagement and recruitment efforts has not been explored extensively in previous literature. The community ambassadors' model has demonstrated impact on increasing community willingness to participate in clinical research [28].

Another recommendation from our study is to boost research visibility through multimodal outreach and messaging. Previous research exploring facilitators to engagement has recommended in‐person community engagement strategies [29, 30]. While our research confirms this recommendation, our findings also highlight that additional outreach strategies, such as social media, may support reaching special populations, such as young people and other hard to reach populations in research [31]. Specifically, our findings call for deploying multiple models of outreach including the formal use of social media by institutions for community engagement and recruitment. While researchers are often worried about fraudulent responses during online recruitment, legitimate participants often suspect online posts from individual social media pages as scams as well [32]. Participants of this study recommended the formal use of social media by institutions which allows for establishing legitimacy and preventing the message being construed as scam. For boosting research visibility, participants recommended encouraging researchers to make people comfortable with the word “research” by raising community awareness about clinical research. Per our research participants, researchers should use every community encounter, be it community outreach for the purposes of education, recruitment, or dissemination of findings, as an opportunity to dispel misinformation about clinical research.

Overall, our findings provide actionable steps for researchers to take before, during, and after research recruitment and overall implementation to enhance relevance of research to the community, rigor, and reach as described in Balazs & Morello‐Frosch's research process framework [24]. Our findings inform research relevance in the recommendations of learning about the community to ask the right questions or conducting research which addresses the problems of importance to the community. In addressing rigor, the recommendations from our participants include diversifying recruitment methods and providing necessary accessibility and accommodations to help researchers reach a diverse sample of participants who mirror the actual community members. Finally, several participants noted the importance of disseminating research findings back to community members, which relates to the reach aspect. This process increases the community's awareness of research, thereby helping in the initiation of a subsequent research project.

Our study comes with some limitations. First, since this is a qualitative study with purposive sampling, our results might not be representative of all other underserved populations in other geographic locations. Further, as is the nature of qualitative research, our findings are hypothesis generating, and the evaluation for actual strategies for increasing research participation among minority populations should be tested using quantitative methods. Despite the limitations, our study comes with major strengths. The participants of our study have unique experiences in that they live in a community affected firsthand by the prominent historical instances of ethical research violations, including the use of Henrietta Lacks' cells for research without consent [33, 34]. Additionally, our participants did not have to be actively engaged in any research study, which allowed us to assess unconflicted community members' perspectives on research participation. Further, our participants comprised community members recruited in community outreach events, people recruited from an online registry, and leaders with influence in their respective communities, facilitating examination of the barriers and recommendations for research participation among underrepresented populations from different viewpoints.

## 5 Conclusion

The underrepresentation of Black and Latino populations in clinical research impacts the generalizability of research and may weaken overall effectiveness of health innovations and other products of those research projects. Identifying strategies to overcome barriers to engagement and diversify participation in research is critical to advance health equity. Our findings provide community‐informed recommendations around four themes: establishing community relationships to foster trust, sustaining engagement through active and transparent communication, boosting research visibility and awareness through multimodal outreach and messaging, and promoting accessibility with person‐centered accommodations. These recommendations can be adapted and tailored to communities across the United States to promote diverse and inclusive clinical research participation.

## Author Contributions

S. Byiringiro, E. Wong, T. Tomiwa, M.G. Vazquez, P. Sheikhattari, C.D. Himmelfarb, H. N. Miller participated in the conceptualization and design of the project. G.C. Bellinger, A. Mezu, E. Wong, T. Tomiwa participated in the formal analysis. S. Byiringiro, G.C. Bellinger, A. Mezu, E. Wong, A. Foster, T. Tomiwa drafted the manuscript. All authors reviewed, edited, and approved the final version of the manuscript.

## Ethics Statement

This study was performed in line with the principles of the Declaration of Helsinki. Approval was granted by the Ethics Committee of the Johns Hopkins University School of Medicine (IRB# 00377197).

## Consent

Participation was voluntary. The consent process involved potential participants reviewing the consent form, engaging with the researcher to ask questions, and signing the informed consent before the actual participation in a FGD.

## Conflicts of Interest

The authors declare no conflicts of interest.

## Supporting information


**Supporting File 1.** Focus Group Discussion Guide.

**Supporting Table 1.** Representative quotes by each stage of the research process to improve Relevance, Rigor, and Reach of the research project.

## Data Availability

All data relevant to the manuscript have been provided in the main article or Supporting Information S2: Table 1.
