# Supplementary material for: From Dialogue to Action: Community Recommendations for Inclusive Research Participation Among Underrepresented Populations
Source: Health Expect. 2025 Jul 30;28(4):e70348. doi: 10.1111/hex.70348 (PMC12310556; doi:10.1111/hex.70348)
Supplement: Supplementary file 1 — Supporting File 1. Focus Group Discussion Guide. [file HEX-28-e70348-s002.docx]

**Facilitator Guide**

***45-60 Minutes***

**Introduction:**

*“Good afternoon! Let me start by telling you how much I appreciate you all for taking part in this conversation. I am from the CONNECT team and my name is [state name]. Our goal in CONNECT is to build a community that helps people learn more about managing their risk for heart disease. We also want to have a pathway for sharing information about research studies on heart disease and make sure that people from all groups can participate in research on heart disease in Baltimore.*

*You have been invited to this conversation today to help us understand what strategies we could use to achieve those goals.”*

This discussion will last between 45-60 minutes, and it will be recorded. Please be sure to speak loudly, and feel free to elaborate on any of your points. Our discussion will cover four key topics:

1. General participation in research,
2. Engagement in research,
3. Connecting with communities about research participation, and

If any question is not clear to you, please feel free to ask me to explain it. The information you provide is invaluable and will be used to shape and inform this project.

**Consent (*Facilitator should read this section as-is):***

*“Before we get started, we need your consent to participate in this discussion. I will review the informed consent with you now.”*

*[read through the consent]*

[Page 6] Section 13 asks for your permission for our research team to contact you in the future about other studies – this is an optional part of our main study, and you can still participate in the main study even if you sign “no” here. [Page 6] Section 14 asks if you would like to participate in the main study, CONNECT. You are free to stop participating in this study at any time and your decision to take part or not take part will not affect your relationship with the researchers, the sponsoring organization, or anybody associated with this study. Your signature here means that you understand the information we went through in this form, that you have had a chance to ask questions, and that you agree to join this study.

We will give you a copy of this consent form. When you are ready, please provide your signature *[on the line below]*, your *[name in print]*, and today’s *[date and time]*. Thank you.

**Confidentiality Statement & Group Norms (Facilitator should read this section as-is):**

Before we begin, I want you to know that this discussion will be recorded and that my colleague(s) [State their name (s)]name) will take notes. As we will be recording, we request that you speak clearly and loudly.

*We really value your thoughts and opinions and want everyone to feel comfortable in space together. Because of this, we have some assurances to give and a few ground rules to guide our conversation.*

1. First, we would like to assure you that all information that you provide us will never be used for anything else other than research purposes, and it will never be given to anyone outside of our research team unless you give us your explicit permission to do so.
2. We kindly request that you not talk about any of the information shared during this gathering to individuals who were not in attendance. This includes any information that could be used to personally identify other people who are here. Besides individuals who are part of the research team, please do not record (audio or video), copy, or share any of what is being discussed here.
3. To maintain your confidentiality, please use only your first name to identify yourself when you speak.
4. To minimize distractions, please turn off your cell phone or put your ringer on silent.
5. [*For Zoom FGDs]* If we get disconnected during this meeting, we will attempt to restart the meeting and call within 2 minutes. If you do not hear from us, please rejoin the meeting using this Zoom link.
6. We would like to hear everyone’s ideas during our discussion, so it is important that only one person is talking at a time and that each person gets equal opportunity to speak. Also, please wait for the previous speaker to finish speaking before you share your thoughts.
7. Please be respectful towards the opinions and ideas of others throughout the discussion.
8. We have a certain amount of time allotted for each question.  If we run out of time on a question, we may need to move on to the next question.  If you have more that you would like to say, please write down your additional comments and we will review these in the end.
9. Please note that you do not have to answer any question that makes you uncomfortable or that you wish not to answer. You do not have to give any reason for not answering any question.
10. You are free to stop participating in this research project at any time. Your decision to take part or not will not have affect your relationship with the researchers, the sponsoring organization, or anybody associated with this study.

If you have any questions or concerns about the privacy of this research or any other aspect of it, please do not hesitate to let me or the other members of the research team know.

Thank you for agreeing to do this.

# **CORE QUESTIONS**

**TOPIC 1: Participating in Research (15 mins)**

**Facilitator:** We will begin with the first topic, “Participating in Research.” In this section, we will discuss your thoughts, feelings, and hesitancies about participating in research. Please feel free to also bring up any experiences related to participation in research, generally. When we say participation, we are referring to joining a research study.

**Question 1: What do you think about the idea of taking part in research?**

*Probe 1*: There are different types of research studies, do you know some of these different types?

*Probe 2*: What should research teams do to be sure participants understand what they are being asked to do?

**Question 2: In your opinion, what are the reasons people decide to participate in research?**

*Probe 1*: How do you think individual and cultural differences play a role in people taking part in research?

*Probe 2*: What personal beliefs or experiences may influence someone’s decision about joining a research study?

**Question 3: In your opinion, what are the biggest reasons people do not take part in research?**

*Probe 1*: What might make it challenging for someone to participate in research?

*Probe 2*: What are some hesitancies, personal beliefs or experiences people might have that make them nervous to join a research study?

**TOPIC 2: Engagement in Research (15 min)**

**Facilitator:** “We will begin with the second topic, ‘Engaging in Research’. In this section, we will discuss ways that researchers can share information about clinical research and invite people to join research studies. When we use the word engagement, we are describing getting a person involved in research.”

**Question 1: Getting people involved in research studies is one of the first things that needs to be done in research. For example, you might reach out to a person to tell them about a research study and invite them to participate. What are some ways you think we can do a better job at getting people to participate in research?**

*Probe 1:* What are some resources that you know of for learning about or signing up to participate in research?

*Probe 2*: What information is important for research teams to share to get people interested in the study?

*Probe 3*: What are ways research teams can communicate why research is important to potential volunteers?

**Question 2: It is also important to keep individuals involved in research over time, such as during the research study. What are some ways you think we can do a better job at getting people to stay engaged in a study after joining?**

*Probe 1*: In your opinion, what do you think would motivate someone to stay engaged? For example, being paid for their participation.

*Probe 2:* What can research teams offer to make participating easier? For example, offering free parking or virtual visits.

**TOPIC 3.** **Connecting with communities about research participation (15 min)**

**Facilitator:** “Now we would like to ask a few questions about ways to reach out to people in the community to invite them to participate in research. These strategies could mean designing recruitment materials to be more meaningful to someone’s culture or sharing information at community events.” **(10 minutes)**

**Question 3: What are your views about researchers attending community events or working with community organizations to connect individuals to research opportunities and share information about research?**

*Probe 1:* One idea our team has is to support individuals in trusted spaces, such as beauty salons and churches, to help raise awareness of research opportunities. The people sharing the information would be individuals who already work in or are connected to those spaces, such as the hair stylist. What do you think of this idea?

*Probe 2:* What has been your experience in receiving or finding research opportunities while in the community. For example, while at church or at a local market?

*Probe 3:* What are some benefits when researchers come to the community to talk to people about research, encourage people in the community to take part in research, and for staying connected to people who join over time? What are some downsides?

*Probe 4:* Can you share some ideas about how researchers can better share information about research to Women and Latinx and Black communities in Baltimore City?

**Question 4:** **What ways or resources do you know of in Baltimore City that can help people find information about ongoing research studies?**

*Probe 1*: If you do not know of any, what are some ways or resources that you think could help people find this information on research studies?

*Probe 2*: What types of resources do Baltimore City residents use to stay informed about research opportunities? What other resources would be helpful to engage community members in research?

**Facilitator: "**Now we would like to ask about ways to reach out to people to participate in research through digital or online methods. For example, this could be by contacting someone through healthcare accounts they have with their providers. This could also include reaching people through online advertisements, social media, text messages, and email.”  **(5 minutes)**

**Question 5: What do you think about using digital tools to reach people who want to take part in research?**

*Probe 1*: Have you ever received information about research studies through digital tools like text messages, emails, and/or websites? Can you share your experience?

*Probe 2*: What are some reasons someone might use digital tools to find research opportunities? Why not?

*Probe 3*: How can these tools be used to support individuals in finding and participating in research studies?

**Synopsis (5 mins)**

1. *Do these themes & descriptions capture your own experiences?*
   1. *Do you agree or disagree with any touchpoints described, or how they are described?*
   2. *Would you change any of the words or descriptions?*
2. *Is anything missing?*
   1. *Should anything be added or removed?*

**Guía del moderador**

***45‑60 minutos***

**Introducción:**

*“¡Buenas tardes! Quisiera comenzar diciéndoles lo mucho que les agradezco su participación en esta conversación. Soy del equipo de CONNECT y me llamo [indique el nombre]. Nuestro objetivo en CONNECT es construir una comunidad que ayude a las personas a aprender más sobre la gestión del riesgo de padecer enfermedades cardiovasculares. E***nfermedad cardiovascular incluye muchas afecciones diferentes que afectan al corazón, como la insuficiencia cardíaca, la arteriopatía coronaria (estrechamiento de los vasos sanguíneos que llevan la sangre al corazón) y los ritmos cardíacos anormales.** *También queremos tener una vía para compartir información sobre los estudios de investigación sobre enfermedades cardiovasculares y asegurarnos de que personas de todos los grupos puedan participar en la investigación sobre enfermedades cardiovasculares en Baltimore.*

*Los invitamos a esta conversación de hoy para que nos ayuden a comprender qué estrategias podríamos utilizar para alcanzar esos objetivos”.*

Este debate durará entre 45 a ‑60 minutos y será grabado. Asegúrese de hablar fuerte y desarrolle cualquier punto que desee. Nuestro debate abarcará cuatro temas clave:

1. participación general en investigación clínica;
2. compromiso con la investigación;
3. contactar a las comunidades acerca de participar en investigación clínica;
4. recibir información sobre  *enfermedades cardiovasculares*.

Si alguna pregunta no le resulta clara, no dude en pedirme que la explique. La información que nos proporcione es muy valiosa y se utilizará para dar forma e inspirar este proyecto.

**Consentimiento *(el moderador debe leer esta sección tal cual está):***

*“Antes de comenzar, necesitamos su consentimiento para participar en este debate. Ahora revisaré el consentimiento informado con ustedes”.*

*[lea el consentimiento]*

[Página 6] En la sección 13 se le pide permiso para que nuestro equipo de investigación se ponga en contacto con usted en el futuro por otros estudios. Se trata de una parte opcional de nuestro estudio principal, y puede participar aunque firme “no” aquí. [Página 7] En la sección 14 se le pregunta si desea participar en el estudio principal: CONNECT. Puede dejar de participar en este estudio en cualquier momento, y su decisión de participar o no participar no afectará su relación con los investigadores, la organización patrocinadora ni ninguna persona asociada a este estudio.

Su firma aquí significa que ha comprendido la información que le hemos proporcionado en este formulario, que ha tenido la oportunidad de hacer preguntas y que está de acuerdo en participar en este estudio.

Le entregaremos una copia de este formulario de consentimiento. Cuando esté listo, firme *[en la siguiente línea]*, escriba su *[nombre* *en imprenta]* y escriba *[la hora y el día]* de hoy. Gracias.

**Declaración de confidencialidad y normas del grupo (el moderador debe leer esta sección tal cual está):**

Antes de empezar, quiero que sepan que este debate será grabado y que mis colegas [indique sus nombres] tomarán notas. Como vamos a grabar, les pedimos que hablen claro y fuerte.

*Valoramos mucho sus ideas y su opinión, y queremos que todos se sientan cómodos en el espacio común. Por ello, queremos garantizar algunas cosas y tenemos algunas reglas básicas para guiar nuestra conversación.*

1. En primer lugar, nos gustaría asegurarle que toda la información que nos proporcione nunca se utilizará para nada que no sean los fines de la investigación y que nunca se le dará a nadie fuera de nuestro equipo de investigación, a menos que nos dé su permiso explícito para hacerlo.
2. Le pedimos que no hable de la información compartida durante esta reunión con personas que no hayan asistido. Esto incluye cualquier información que pudiera utilizarse para identificar personalmente a otras personas presentes. Excepto las personas que forman parte del equipo de investigación, no grabe (en audio ni en video), copie ni comparta nada de lo que se está discutiendo aquí.
3. Para mantener la confidencialidad, use solo su nombre (sin apellido) para identificarse cuando hable.
4. Para minimizar las distracciones, apague el celular o póngalo en silencio.
5. *[Para los grupos de debate por Zoom]* Si nos desconectamos durante la reunión, intentaremos reanudarla y llamaremos en 2 minutos. Si no tiene noticias nuestras, vuelva a unirse a la reunión utilizando este enlace de Zoom.
6. Nos gustaría escuchar las ideas de todos durante nuestro debate, por lo que es importante que solo hable una persona a la vez y que cada persona tenga la misma oportunidad de hablar. Además, espere a que la otra persona termine de hablar antes de compartir sus ideas.
7. Sea respetuoso con las opiniones e ideas de los demás durante todo el debate.
8. Disponemos de cierta cantidad de tiempo para cada pregunta. Si se nos acaba el tiempo para una pregunta, es posible que tengamos que pasar a la siguiente. Si tiene algo más que le gustaría decir, escriba sus comentarios adicionales y los revisaremos al final.
9. Tenga en cuenta que no está obligado a responder ninguna pregunta que le incomode o que no desee responder. No tiene que dar ninguna razón por no responder las preguntas.
10. Puede dejar de participar en este proyecto de investigación en cualquier momento. Su decisión de participar o no participar no afectará su relación con los investigadores, la organización patrocinadora ni ninguna otra persona relacionada con este estudio.

Si tiene alguna pregunta o duda sobre la privacidad de esta investigación o cualquier otro aspecto de la misma, no dude en comunicármelo a mí o a los demás miembros del equipo de investigación.

Gracias por aceptar participar en este estudio.

# **PREGUNTAS PRINCIPALES**

**TEMA 1: Participación en la investigación (20 minutos)**

**Moderador:** “Comenzaremos con el primer tema: participación en la investigación. En esta sección, hablaremos sobre sus pensamientos, sentimientos y dudas sobre participar en un proyecto de investigación. No dude en mencionar también cualquier experiencia relacionada con participar en un proyecto de investigación en general. Cuando decimos *participación*, nos referimos a unirse a un estudio de investigación”.

**Pregunta 1: ¿Qué le parece la idea de participar en una investigación?**

Sondeo 1: Existen diferentes tipos de estudios de investigación, ¿conoce algunos de estos diferentes tipos?

Sondeo 2: ¿Qué deben hacer los equipos de investigación para asegurarse de que los participantes entienden lo que se les pide que hagan?

**Pregunta 2: En su opinión, ¿cuáles son las razones por las que las personas deciden participar en una investigación?**

Sondeo 1: ¿Cómo cree que influyen las diferencias individuales y culturales en la participación de las personas en la investigación?

Sondeo 2: ¿Qué creencias o experiencias personales podrían influir en la decisión de alguien de participar en un estudio de investigación?

**Pregunta 3: En su opinión, ¿cuáles son las principales razones por las que la gente no participa en una investigación?**

Sondeo 1: ¿Qué podría dificultar la participación de alguien en una investigación?

Sondeo 2: ¿Qué dudas, creencias personales o experiencias pueden hacer que las personas se sientan nerviosas a la hora de participar en un estudio de investigación?

**TEMA 2: Compromiso con la investigación (15 minutos)**

**Moderador:** “Comenzaremos con el segundo tema: compromiso con la investigación. En esta sección analizaremos las formas en las que los investigadores pueden compartir información sobre la investigación clínica e invitar a las personas a participar en los estudios de investigación. Cuando utilizamos la palabra *compromiso*, describimos cómo hacer que una persona se una a la investigación”. **(20 minutos)**

**Pregunta 1: Hacer que las personas se unan a los estudios de investigación es una de las primeras cosas que hay que hacer en las investigaciones**. **Por ejemplo, puede contactar a una persona para hablarle de un estudio de investigación e invitarla a participar. ¿De qué manera cree que podemos mejorar la forma en que hacemos que las personas participen en la investigación?**

*Sondeo 1:* ¿Qué recursos conoce para informarse o inscribirse para participar en una investigación?

Sondeo 2: ¿Qué información es importante que compartan los equipos de investigación para que la gente se interese en el estudio?

Sondeo 3: ¿De qué forma los equipos de investigación pueden comunicar por qué la investigación es importante para los posibles voluntarios?

**Pregunta 2: También es importante mantener a las personas involucradas en la investigación a lo largo del tiempo, por ejemplo, durante el estudio de investigación. ¿De qué manera cree que podemos mejorar la forma en que hacemos que las personas sigan participando en un estudio después de unirse a él?**

Sondeo 1: En su opinión, ¿qué motivaría a alguien a seguir participando? Por ejemplo, recibir una remuneración por su participación.

*Sondeo 2:* ¿Qué pueden ofrecer los equipos de investigación para facilitar la participación? Por ejemplo, ofrecer aparcamiento gratuito o sesiones virtuales.

**TEMA 3:** **Contactar a las comunidades acerca de participar en la investigación (15 minutos)**

**Moderador:** “Ahora nos gustaría hacer algunas preguntas sobre las formas de llegar a las personas de la comunidad para invitarlas a participar en la investigación. Estas estrategias podrían ser cosas como diseñar materiales de reclutamiento para que sean más significativos para la cultura de alguien o compartir información en eventos de la comunidad”. **(10 minutos)**

**Pregunta 3: ¿Qué opina de que los investigadores asistan a eventos comunitarios o que trabajen con organizaciones comunitarias para contactar a las personas por oportunidades de investigación y compartir información sobre la investigación?**

*Sondeo 1:* ¿Cuál ha sido su experiencia con respecto a recibir o encontrar oportunidades de investigación mientras se encontraba en la comunidad? Por ejemplo, en la iglesia o en un mercado local.

*Sondeo 2:* ¿Cuáles son algunas de las ventajas de que los investigadores vayan a la comunidad para hablar con la gente sobre la investigación, animar a las personas de la comunidad a participar en la investigación y para mantenerse en contacto con las personas que se unen a lo largo del tiempo? ¿Cuáles son algunas desventajas?

*Sondeo 3:* ¿Puede compartir algunas ideas sobre cómo los investigadores pueden compartir de una manera mejor información sobre la investigación con las mujeres y las comunidades latinas y negras de la ciudad de Baltimore?

**Pregunta 4:** **¿Qué formas o recursos conoce en la ciudad de Baltimore que puedan ayudar a las personas a encontrar información sobre los estudios de investigación en curso?**

Sondeo 1: Si no conoce ninguno, ¿cuáles son algunas formas o recursos que cree que podrían ayudar a las personas a encontrar esta información sobre estudios de investigación?

Sondeo 2: ¿Qué tipo de recursos utilizan los residentes de la ciudad de Baltimore para mantenerse informados sobre las oportunidades de investigación? ¿Qué otros recursos serían útiles para implicar a los miembros de la comunidad en la investigación?

**Moderador:** “Ahora nos gustaría preguntar sobre las formas de llegar a las personas a través de métodos digitales o en línea para que participen en la investigación. Por ejemplo, se podría hacer contactando a la gente a través de las cuentas de atención médica que tienen con sus proveedores. Esto también podría ser llegar a la gente a través de anuncios en línea, redes sociales, mensajes de texto y correo electrónico”. **(5 minutos)**

**Pregunta 5: ¿Qué opina sobre el uso de herramientas digitales para llegar a las personas que desean participar en la investigación?**

Sondeo 1: ¿Alguna vez recibió información sobre estudios de investigación a través de herramientas digitales, como mensajes de texto, correo electrónico o sitios web? ¿Puede compartir su experiencia?

Sondeo 2: ¿Por qué la gente podría utilizar herramientas digitales para encontrar oportunidades de investigación? ¿Por qué no?

Sondeo 3: ¿Cómo pueden utilizarse estas herramientas para ayudar a las personas a encontrar estudios de investigación y participar en ellos?
