# Supplementary material for: From Dialogue to Action: Community Recommendations for Inclusive Research Participation Among Underrepresented Populations
Source: Health Expect. 2025 Jul 30;28(4):e70348. doi: 10.1111/hex.70348 (PMC12310556; doi:10.1111/hex.70348)
Supplement: Supplementary file 2 — Supporting Table 1. Representative quotes by each stage of the research process to improve Relevance, Rigor, and Reach of the research project. [file HEX-28-e70348-s001.docx]

**Supplementary Table 1. Representative quotes by each stage of the research process to improve Relevance, Rigor and Reach of the research project.**

| **Step** | **Quote** |
| --- | --- |
| Research goals | I think one of the things that we need to begin to look at is, we need to first learn our culture, learn the people who we'll be working with. Find out a little bit about the community, what their needs are, and what their concerns are… Trust on being very honest, changing language, and making sure you are culture competent, that you know the community that you're working with. (Participant 4, FGD #2) |
| Research goals | I think we want to get to the point with the word research that we're not whispering it. We're not afraid of it and we understand it. We only can do that by talking about why we research, what is research, and get it to that level where it's an open conversation—before we even invite you to be a part of a study. (Participant 9, FGD #2) |
| Research goals | You really do need to research your community, understand the people that you are working with. I do believe that cultural competency plays a major part. (Participant 4, FGD #1) |
| Research goals | I think when you go out in the community and work with the leaders, I think you need to have a concept, an idea, of what their focus is, their particular topic is, at that particular, given time. You can't come with a research study or a topic, if you will, that you want to introduce to the community. Again, it goes back with building relationships. (Participant 8, FGD #2) |
| Research goals | I think to improve, I think that you do need to make efforts to engage the community long before you’re asking them to participate in a research. (Participant 2, FGD #1) |
| Research goals | ...building those relationships, even when you aren't recruiting for a study, so that you can go into the community... I think that's hard because the sort of the primary focus of researchers is to kind of collect the data, evaluate it, and then get it sort of published for more funding. Right? That's sort of like the cycle there. (Participant 7, FGD #1) |
| Research goals | When we get into a community, we need to do our research, need to understand what people need, what they like, what they understand, and what they're looking for; not to come there already deciding you need this, or you need that, or you should need this. We only can do that by listening... We're listening to what people are saying. We might not be talking about our study at that time, but we can capture what is their interest and what their needs are in the community. (Participant 9, FGD #2) |
| Research goals | I would probably say that getting people to see that the research will benefit them and their communities in a major way—instead of just being for the institution—you're going to help our communities be better health-wise and be better mental health-wise. Or whatever the result of the study is, that the community will benefit from the study in some major way, instead of just a publication that the university will be able to use. (Respndent 4, FGD #2) |
| Study design | ...community based participatory research, so we lead the research as a community because we know what we need. Then the researchers come alongside of us to work, collaborate with us, to help us to get results that we know—we know where it's going. We know what outcomes we want. Because we're developing them. (Participant 5, FGD #1) |
| Data collection | Providing culturally tailored approaches... The recruitment process should be customized. That also goes along with how you're going to communicate to the individuals, and making sure that you are culturally sensitive and that the research is relevant to that particular targeted population. (Participant 5, FGD #2) |
| Data collection | I think you can become more visible in the community, and just talk about it. Just talk about it and let it permeate in the air. Talk about it. How important it is. Would you like to be a part? I think we need to have more conversations. We need to have more conversations and just talk about it more. Make it a household name. Make it feel like it's part of what we should do to help out. (Participant 2, FGD #2) |
| Data collection | The way a researcher comes into a community will affect the way—the decision whether somebody will join. You can't go into certain communities and just say, "Oh, we're looking for somebody to sign up," and expect people to sign up. Sometimes, you need a mediator or a point person, like, say, a religious leader or something to come or a community leader or community organizer to come and say, "Oh, this is Soandso, and this is what they're trying to do." I think that plays a role in it as well, how they come into the community. (Participant 9, FGD #3) |
| Data collection | Black churches. Higher education institutions teach advocacy, but they don't practice advocacy. If you learn advocacy, you know that there are structures in communities. Especially in the black community, you can't just walk in and do stuff. You have to go through some channels. Black churches. She mention black fraternities and sororities. These are avenues for you to get the people that you looking for, but you always bypass them and go to the people themselves... You have to know your community. You have to go through the channels in the community to know make it work. Create a flier. Give it to the health unit in the church. Let them distribute. It's coming from the church. Let the preacher preach, on one Sunday, about whatever you doin'. They would do it, willingly. (Participant 9, FGD #6) |
| Data collection | By participating, it allows us to contribute to the development of possible better healthcare treatments, interventions; and also, I believe, that representation matters. I think having participation and being a part in a diverse research group, it provides researchers with accurate and inclusive data. That's where it is. (Participant 5, FGD #2) |
| Data collection | 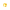Also understanding the nuances of the communities and meeting them where they actually gather, not treating them as a monolith because you tend to lose different sectors of that particular group. (Participant 9, FGD #3) |
| Data collection | I think relationship buildin' is very, very important in this field. People can spread your information to other people. You need to build a relationship with a core group of people that have either participated in your research or know about the type of work that you do that they can spread to other people. Again, a impersonal thing doesn't work. (Participant 9, FGD #6) |
| Data collection | ...use your community advocates, your community leaders. We know best who's in our community. They trust us. We're able to talk to them. Inform your community leaders to inform the community residents. Again, it goes back to talk about it more, have open communications, and open dialogues with your community leaders. If you can convince a community leader, you can convince the entire community. (Participant 2, FGD #2) |
| Data collection | I think that we would feel more comfortable if we had an assistant guiding us through the process. It could be someone who’s gone through the process, or a person who is part of the research team, but that has enough knowledge to communicate to the person about what is going to happen, so the person feels at ease. (Participant 3, FGD #5) |
| Data collection | This was just a thought too, but like also training, you know, the local community to do the follow up and the engagement too. So like really, bringing a holistic approach to it. (Participant 1, FGD #1) |
| Data analysis | Something that I always see is that when you do a study, you don’t inform, like, there is also a group of Americans that are being studied. It’s only Latinos. So, as Latinos, they make you think that they’re only studying us. Do you understand what I’m saying?  Being able to inform that there is also a group of North Americans that are in the study to see X things, and then there is a group of Latinos that are there for the same thing, but in a different area, whether because of the language or because of cultural factors, or other factors. (Participant 3, FGD #5) |
| Dissemination of results | I think that they should try to give us information about what they did to us and tell us if it worked or not... Otherwise... you don’t even know what happened. So, we don’t go back because we’re afraid. Yes, we’re thinking that they’re experimenting on us, so information, especially because if you don’t know anything, we don’t go back. (Participant 1, FGD #5) |
| Dissemination of results | They sometimes...they came, they work with us, and then after two years that we’re working together, they left. We get the pay, but we never see them anymore. So, we don’t know if that was successful or they did something, we don’t know. (Participant 5, FGD#1) |
| Dissemination of results | But if they don’t say nothing, we will never know if ...what we did was good or not. So that is why some people don’t want to participate. They say, “Oh no. We just stayed there for one year and nothing happened.” (Participant 5, FGD #1) |
| Dissemination of results | ...it seems to me that researchers spend a lot of time and money presenting their findings around the world, and the least they could do is go to the local school in that community and present their findings to the people that they used in their research. I think that that really goes a long way for inclusivity. Also it's just—it feels like it's like human respect there, and gratitude for the service of being a part of the study. (Participant 1, FGD #2) |
| Dissemination of results | It’s so important, what you said about the follow-up. Havin’ some continuity, I think it’s like—you’re like okay, I did all of that, I made my way down there, I took my time which is valuable. It may not necessarily be monetary to them, but your time is valuable. I’m gonna come so tell me how my visit—what’s the next step? How did I make a difference? How can I make a difference? If you just drop it, what’s the point? It’s like it’s another little drop in the sea. You know? Like what difference does it make? (Participant 3, FGD #7) |
| Dissemination of results | You send me a email saying, "You know what, because of your contribution, this is what happened." (Participant 3, FGD #6) |
| Dissemination of results | There's always an abstract sheet that they give out to people. Send me one of them as a person that contributed to the research. (Participant 9, FGD #6) |
| Dissemination of results | ...also in developing that reporting, to have that participant a part of that, so they can see their voice, their name, in the documentation. I think that gives more ownership and more partnership. We're not just hearing your words or your voice but also hearing mine. (Participant 4, FGD #2) |
| Dissemination of results | One mistake that we make is that we do the research. It's completed, but nothing comes to me, as a participant. I don't even know—you interviewed me for the research, but I don't know the outcome. Send me the summary sheet so I can figure out what you got out of me. (Participant 9, FGD #6) |
| Dissemination of results | I am going to inform you how you will participate in the study, but I will also inform you how it concluded. How it ended, and if there were results or not, whether positive or negative ones. (Participant 3, FGD #5) |
| Evaluation of process | After conducting a study, and obtaining certain results, we could have an interview with the person or people involved asking them about their experience. In that way, you invite other people to participate in certain studies. (Participant 3, FGD #5) |
|  | how does the community benefit? It often feels like you come in for three years, you gather your data, and then you leave, and there's no—there's no long standing impact of a positive nature on the community. I think that that is often troubling to the people that I work with. (Participant 7, FGD #2) |
| Dissemination of results | Yes, I totally, totally agree... taking a collaborative approach. Bringing them along, letting them know that you're definitely a part of this research. Like you say... just even with—from a personal standpoint, keeping in touch with them, but keeping—I think you can certainly keep them engaged that way, to continue to communicate with them, include them in everything that you find; any updates, anything that you plan to do going forward, but just to make sure that you continue to keep the communication going. I think the takeaway that I've got—and trust is a big word. I think it's been used on all sides of the spectrum. When we talk about research, open communication, and definitely relationship building, that is very key. They are all very key when doing research with community members. (Participant 8, FGD #2) |
|  | A lot of our communities they participate with, however the situation is, in these research studies and then after the research is over, POOF – they’re gone. And then it’s like, that is really unfortunate because we still have community members who maybe need continued healthcare access, who you know, who need all these types of support. The way I view it is if certain research was done with a community to “improve” the quality of life, why are we not focusing on it long term? Not just in that short term situation to kind of get the results and everything. You know, I think that’s a really big thing. For example, my mom, her opinion has always been, like “what do I get after that?”. Not more so like where she is looking for more incentives anything but like “You're just gonna drop me and forget me and then I’m gonna see you on tv”. (Participant 6, FGD #1) |
|  | But they come in and ask you to participate and after that... you never see them anymore. And what value has it brought to the community? ...I would say that for a long time, people have not seen that there was a benefit to them or their community. (Participant 2, FGD #1) |
|  | I think in all the years I've done outreach or community work, I've only ever been approached by one researcher in the community and I really appreciated that. Because instead of just making assumptions or going out, you know, their own way to helping the community, they relied on the experts which were the people working in the community. Community health workers, community members, schools, and I think that is really important to have that representation. Having researchers as part of those types of events, one, builds trust. And they end up becoming something familiar to the community. That way it's not just a random thing or person or group that came to the community. And then the other thing is really, just, having research as part of this builds cultural competency within themselves. You know, that's the big thing that I can go on and on about, the lack of cultural competency even within research, you know. Just really quickly that I was gonna say, I would say, “Latinx” doesn’t necessarily represent the whole community, that term, it's actually very, very new. And not many people know what it is. Just for reference, Hispanic that term was created in the United States in the eighties. “Latino” came a little bit after that and the “Latinx” is this new thing. So, all of those terms, you know, are really not things we know too well. So, researchers being in the community, they can hear how people actually refer to themselves as opposed to just assuming what they call themselves. (Participant 6, FGD #1) |
|  | there’s a lot of work happening in the public-health space around the community-health-worker model. I think that that is a model that would be worthy of lookin’ at in terms of clinical-trials research and especially with the community engagement and participation because, again, there’s nothing like having a member of the community that understands that community, that’s from the neighborhood, understands the people to come in, be trained on this and that they are a representative of them that community members can go to, ask questions, have a little bit more trust in, have those conversations. That’s just not gonna be the same with the typical research team, the whitecoats. Those would be two things that come to mind for me in terms of just tryin’ to get people to be even more open to the possibility of it all. (Participant 2, FGD #4) |
| Data collection | 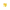I think really, again, doing the research, reaching out to people within the community that you want to work with, and then bring them on to—again, I think collaboration is a great way to better share the research with that targeted population, to collaborate with somebody from that community. (Participant 8, FGD #2) |
|  | I think that people show up, but not just the time. It's gotta be in the long term and as researchers, they should include that in the timeline. Before they start, they should like to start showing up to different community events and you know over and over and over. A commitment. It needs to be a commitment. (Participant 4, FGD #1) |
|  | I think that actually working a little bit more with the leaders and the teams of those organizations can yield fruitful events because they already have the trust of the community. They already have the following. If you’re able to work with those leaders in a way that they can become ambassadors of what it is that you’re lookin’ for, that that may be helpful. (Participant 2, FGD #4) |


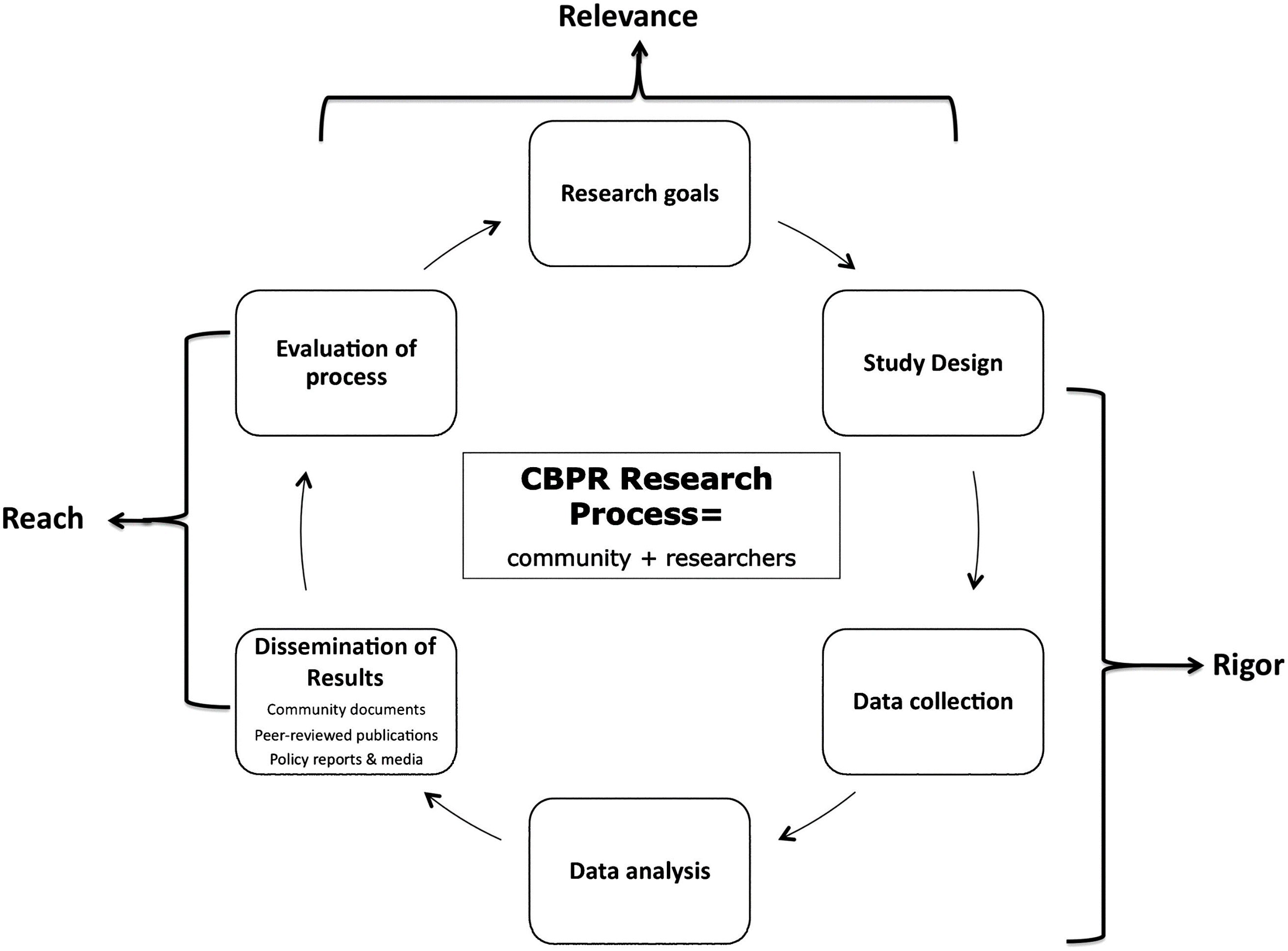


**Reference:** Balazs CL, Morello-Frosch R. The Three R's: How Community Based Participatory Research Strengthens the Rigor, Relevance and Reach of Science. *Environ Justice*. 2013;6(1):10.1089/env.2012.0017. doi:10.1089/env.2012.0017
